# Whose responsibility? Deconstructing global responsible AI from the African lens: a review

**DOI:** 10.1007/s43681-026-01418-3

**Published:** 2026-10-06

**Authors:** Damian Eke, Makuochi Nkwo, Ibifuro Robert Jaja, Muhammad Adamu, Simisola Akintoye, Michael Ugom, Sarah Kiden

**Affiliations:** 1 School of Computer Science, University of Nottingham, Nottingham, UK; 2 School of Computing and Mathematical Science, Faculty of Engineering and Science, University of Greenwich, London, UK; 3 Centre for Philosophy and Artificial Intelligence Research {PAIR}, University of Erlangen-Nuremberg, Erlangen, Germany; 4 Northumbria School of Law, Northumbria University, Newcastle upon Tyne, UK; 5 Centre for Environmental Management and Green Energy (CEMAGE), University of Nigeria, Nsukka, Nigeria; 6 School of Electronics and Computer Science, University of Southampton, Southampton, UK

**Keywords:** Responsible AI, Africa, Deconstruction, Multi-vocal literature, Epistemic injustice, Decoloniality

## Abstract

This paper uses the theory of decoloniality to critically examine how African scholars have interrogated the dominant, global narratives surrounding responsible Artificial Intelligence (AI). Through a multivocal literature review, the paper reveals a range of critical perspectives that expose systemic exclusions and imbalances in the current responsible AI discourse. Key themes identified include the marginalisation of African epistemologies, values, and principles; the absence of representative African datasets, lack of locally grounded regulatory frameworks; the limited inclusion and or availability of African expertise and funding; and the persistent power asymmetries and coloniality embedded in global responsible AI ethics and governance. The review highlights emerging efforts towards reconstructing responsible AI from an African contextual lens. Central to this constructive turn is the proposal of a decoloniality framework that puts emphasis on African knowledge systems, reclaims agency in AI development, and reimagines responsible AI in ways that are contextually grounded, pluriversal, and socially acceptable. This review not only contributes to critical AI scholarship but also charts pathways for more inclusive and equitable AI futures on the continent.

## Background

Responsible Artificial Intelligence (AI) is not a novel concept but rather an extension of long-standing ethical discourse that has followed advancements in technologies throughout history [1]. The ethics of technology have been debated since the invention of tools and machines, from the moral considerations of industrialisation in the nineteenth century, the ethical concerns raised by the development of computers and the Internet in the twentieth century to the risks of nanotechnology. As AI has evolved, the debates have persisted and are rooted in deeper philosophical questions about the nature of human agency, the consequences of automation, and the balance of power between creators and users of technology. Indeed, AI ethics is deeply connected to a broader historical dialogue on the risks and consequences involved in technological innovations and the values embedded in them. Its origins are connected to what is often referred to as ‘Computing Ethics’, ‘Information Ethics’ and ‘Cybernetics’, all of which are parts of Applied Ethics discourse. The rationale for most of the early arguments for AI ethics was based on fundamental concerns/risks related to unfair bias, and human rights including privacy breaches and security. Since its earliest formulations, there has been an agreement that the AI lifecycle (i.e., ideation through to design, development, deployment and maintenance) needs to be responsible. What has followed the initial conceptualisation of responsible AI is the subsequent institutionalisation of ‘‘what constitutes ‘ethical AI’ or ‘responsible AI’ and which ethical requirements, technical standards and best practices are needed for its realisation’’ [2]. However, in recent times, the foundational principles, approaches, nature and operationalisation of responsible AI have come under growing critique. We align this to the decoloniality theoretical lens [3, 4] which is what this paper refers to as the ‘deconstruction’ phase of the history of responsible AI. In this phase, many scholars are re-evaluating and reimagining responsible AI, particularly as it relates to issues of power, inclusivity, and justice. However, this deconstructive turn has a ‘constructive’ phase.

This paper is motivated by the assumption that the discourse of responsible AI in Africa, mainly focuses around the phase of deconstruction—challenging the underlying preconceptions. If such an assumption could be substantiated, therefore, this paper seeks to map the prevalent arguments of this deconstruction stage, arguing that such an intellectual project is not enough to ensure emerging technologies work for the public. Our awareness of the inherent coloniality, injustice and power imbalance in AI design, development, and deployment which characterise the deconstructive stage is not enough; our meticulous deconstruction of global perspectives of responsible AI is relatively insufficient. Therefore, this paper asks the question; *What are the assumptions inherent in global understanding of responsible AI and how can the critical questioning of these assumptions lead to a reimagined meaning of the concept?*

In answering this question, this paper suggests a framework centred on decoloniality [5, 6]. This paper calls for the refocusing of emphasis from deconstruction to construction. The practice and governance of AI in Africa requires a construction of responsible AI that is clear to all relevant stakeholders; from designers, developers, engineers, deployers, academic researchers, policymakers and citizens alike. For responsible AI and governance scholars, this may provide a new avenue for contributing to the wider societal discussion of value alignment in AI. A key contribution of this paper is the argument for decoloniality which can fundamentally shape the design, ethics and governance of AI in African contexts.

This paper is structured as follows: First, we present an overview of the global landscape concerning responsible AI and trustworthy AI initiatives in Africa. Subsequently, we engage with the decoloniality theory to inform our interpretation of the current responsible AI (RAI) landscape from African perspectives. We then detail our methodological choices and research processes. Following this, we present findings that show the deconstruction of prevalent RAI concepts identified in the literature. The paper concludes with a critical discussion reflecting the constructive efforts of African scholars and outlining future directions for research and practice.

## Global landscape of responsible

**Fig. 1:**
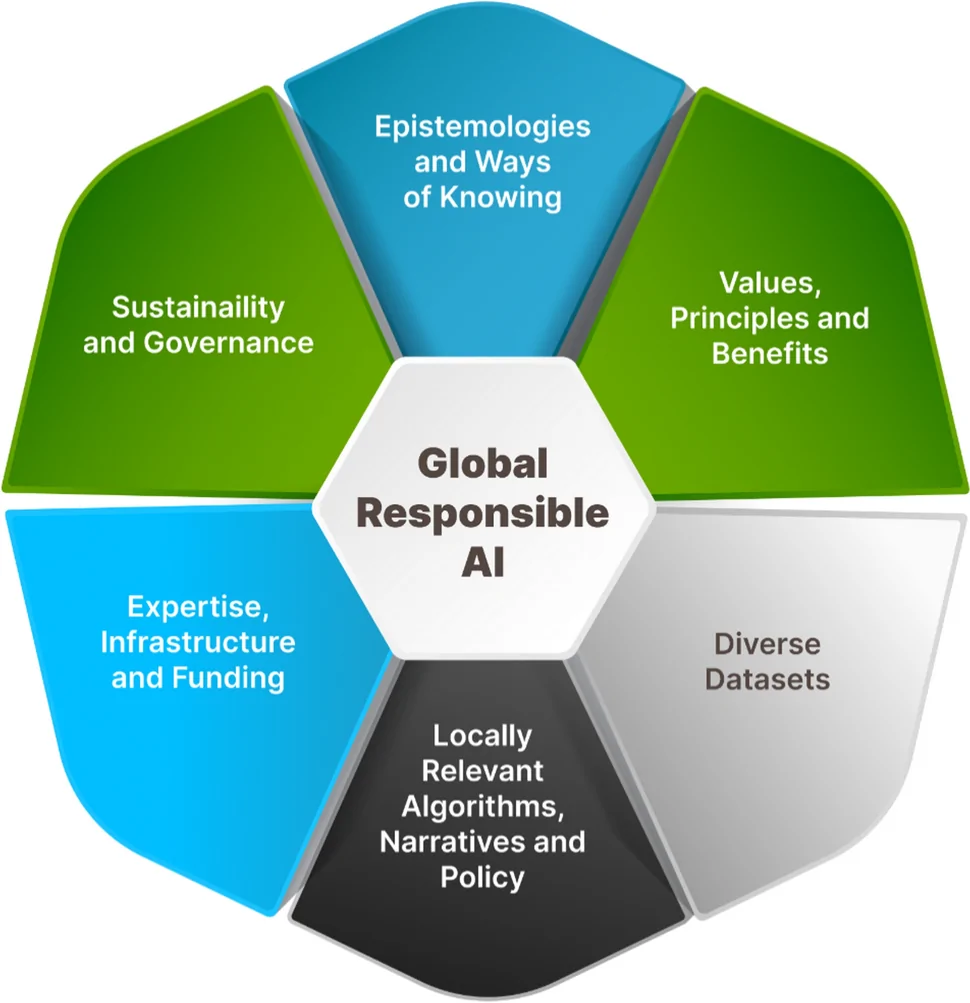
Global responsible AI: Six wedges around a central hub, each naming one dimension of how responsible AI is currently framed in mainstream (Global North) discourse: epistemologies, values, datasets, algorithms/narratives/policy, expertise/infrastructure/funding, and sustainability/governance

Responsible AI frameworks and approaches have emerged worldwide in the past decade, influenced by regional values, political ideologies, and technological capabilities [2, 7, 8]. Responsible AI here encompasses the ethics and governance of AI; the practices and theories aimed at achieving ethically responsible, socially acceptable and legally compliant systems. Responsible AI narratives provide “fundamental theoretical approaches on how everyone involved in the AI lifecycle understands and acknowledges their roles in creating ethical, fair, inclusive, explainable, and accountable AI systems’’ [9]. It is about ensuring that the design, development, and deployment of AI systems are carried out responsively and responsibly as well as ensuring that these systems are sensitive to human and environmental needs and interests. It is now generally understood that AI, like any other emerging technology, is value laden. This means AI systems are embedded with values (like autonomy, justice, responsibility and privacy) and underlined by principles and interests. Oftentimes these values are discussed with preconceptions of shared understanding [10] (Fig. 1). The question is; whose values and whose interests?

**Fig. 2:**
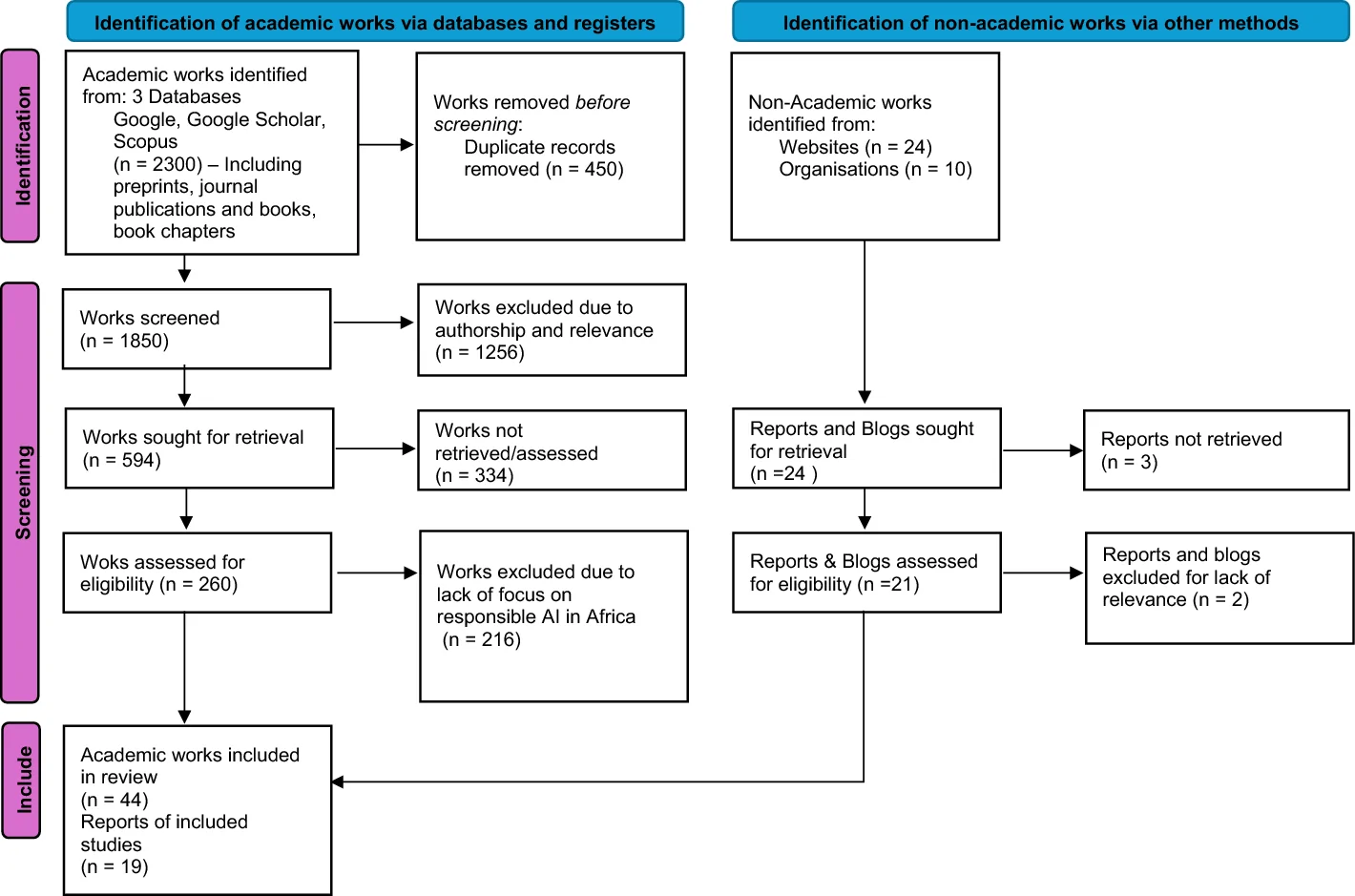
PRISMA flow diagram for the search

The current responsible AI landscape is characterised by narratives, frameworks, principles, values, data and epistemologies developed and driven by institutions and countries in the Global North but often discussed as universal standards (see Fig. 2). Typical issues these frameworks address include bias, discrimination, concerns about security, safety, data privacy, lack of transparency and possible infringements on human rights. However, what counts as ‘harm’, ‘bias’, ‘privacy’ or ‘safety’ is different in different cultural contexts and regions. In the last few years, voices from Africa have arisen to point this out; highlighting the apparent marginalisation or neglect of values, principles, perspectives and contexts in the region. This paper meticulously identifies these views as an intentional deconstruction of the concept of responsible AI which should then lead to a rethinking or reimagination of a global discourse that is sensitive to realities in different regions and contexts.

## Decoloniality and the coloniality of power: a theoretical framework

The theoretical lens underlying our arguments in this paper is decoloniality [3, 11]. Decolonial theory interrogates how supposedly neutral or universal categories are structured by hidden hierarchies and treats critique as a precursor to reimagining rather than an end in itself. This is a theory that focuses on a specific historical structure, coloniality, that produces and sustains the hierarchies this paper is concerned with. It is worth distinguishing here between colonisation and coloniality, and between decolonisation and decoloniality, terms this paper uses precisely rather than interchangeably.

Colonisation (or colonialism) refers to the historical process of direct political, administrative and territorial domination that formal empire exercised over Africa, and which formally ended with the continent's mid-twentieth-century independence movements. Coloniality, by contrast, focuses on what has survived the formal ending of colonialism: the enduring patterns of power, knowledge production and subjectivity that colonial rule established, and that continue to organise global relations long after administrations were handed over. The same distinction holds for decolonisation and decoloniality. Decolonisation was the political and juridical process by which nations achieved sovereignty. Decoloniality is the ongoing critical and epistemic project of undoing coloniality, work that remains necessary precisely because political decolonisation did not, on its own, dismantle the deeper hierarchies of power and knowledge that coloniality sustains. As Wakunuma et al. [5] and Eke et al. [6] argue this is why decoloniality, rather than decolonisation, is the operative term for African AI ethics and governance. African states are formally and politically independent, yet the frameworks, infrastructures and epistemologies that shape AI on the continent remain patterned by coloniality, which is precisely what a decolonial approach to responsible AI must address.

Quijano’s concept of the coloniality of power [12] describes how the racial and geopolitical hierarchies established under colonialism did not end with formal political independence but were reorganised into an enduring pattern of power that continues to structure global relations of labour, knowledge and subjectivity. Quijano identifies three interlocking dimensions of this pattern: coloniality of power. This is about the control over economic and political structures; the coloniality of knowledge or the elevation of Eurocentric rationality as the sole legitimate standard of truth, and coloniality of being which is the classification of colonised peoples as less than fully modern subjects. Applied to responsible AI, the coloniality of power helps explain why the dominant frameworks in this space, the standards and institutions, remain concentrated in the Global North even as AI systems are deployed globally. What presents itself as a universal, technical or neutral standard of "responsible" or "ethical" AI is, on this account, better understood as one regional tradition that has been generalised into a global norm, while other traditions are relegated to the margins [5, 13].

This is where the coloniality of knowledge becomes especially salient. Global AI ethics frameworks tend to assume that concepts such as fairness, accountability, transparency and privacy carry a single universal meaning across contexts. Decolonial theory disputes this on principle, simply because knowledge is always produced from a geopolitical, cultural and historical position. No ethical vocabulary is in fact context-free, including the one that currently dominates global discourse [3]. Walter Mignolo’s account of the "decolonial option" builds on Quijano to argue that the appropriate response is not to establish a rival universal in place of the Eurocentric one, but to "detach" from the colonial matrix of power altogether and open a space for the co-existence of multiple epistemic traditions; pluriversality in place of universality [3, 14]. Our argument that responsible AI in Africa should not seek a new epistemic centre, African or otherwise, but should instead be reimagined as a pluriversal (recognition of multiple views rather than current universal leanings), draws directly on this detachment move.

Furthermore, Sabelo Ndlovu-Gatsheni describes the central task of decoloniality as the pursuit of "epistemic freedom": the right of African scholars, publics and institutions to think, theorise and interpret the world using their own frames of reference, rather than perpetually validating or borrowing frameworks developed elsewhere [4, 11]. Ndlovu-Gatsheni frames this as both a movement to refuse to treat African thought as a parochial supplement to an implicitly Western body of knowledge and the recognition of Western thought as itself one regional tradition among many, rather than the standard against which others are measured. Reading this together with Fricker’s concept of epistemic injustice, which we draw on [15], Ndlovu-Gatsheni’s framing helps explain why African contributions to responsible AI are so often received as commentary on, rather than as a challenge to, dominant global frameworks. Mbembe further argues that coloniality also reflects not only through the extraction of land and labour but through the construction of an archive, the accumulated record of what counts as valid knowledge, from which colonised peoples and their intellectual traditions are largely excluded or included only as objects of study [16].

In sum, this theory situates the growing body of African literature on AI ethics and governance not as a series of isolated complaints but as a coherent decolonial intervention, one in which different contributions address different faces of coloniality, whether of power, knowledge or being, with a common orientation. It also indicates a trajectory: from naming and critiquing coloniality in global AI discourse, toward practical, pluriversal implementations of responsible AI that are accountable to specific African contexts rather than to a single, assumed epistemic centre.

## Methodological approach

This study adopts a Multivocal Literature Review (MLR) approach to explore the emerging landscape of literature on responsible AI in Africa. It synthesises views from formal academic literature and grey literature such as reports, policy documents, media sources, and other contributions from African scholars and practitioners. This approach is especially useful in under-researched domains where peer-reviewed works may be emerging but practice-based knowledge is useful and influential [17–19]. MLR has been used in fields such as software engineering [18–20] information systems [19], cybersecurity [21], and education [22]. These show that although it is not commonly used like other systematic literature reviews, it is an established approach for literature review.

Beyond the combination of evidence-based insights from academic literature and the recent and real-world insights not yet covered by academic studies, MLR improves context sensitivity. In this case, grey literature highlights concerns rooted in African socio-political contexts that are not evident from literature. It also supports reflexivity and encourages critical analysis by revealing tensions between formal and informal discourses. On the other hand, MLR is criticised for the inclusion of grey literature that lacks standardised quality control that can ensure credibility and rigour [22]. They are not systematically indexed like academic databases making identification difficult. In addition, literature from organisations can also contain biased views [ibid]. We acknowledge the above challenges and make clear that our aim is to understand the framing of responsible AI in Africa by diverse sources in Africa. Every framing of responsible AI in this context is useful to build a useful narrative for the emerging ecosystem.

​​The MLR approach reveals a dynamic tension between global normative frameworks and African epistemological realities. Where academic literature tends to offer conceptual critiques, grey literature surfaces emerging innovations, practical concerns, and context-sensitive models. Together, these perspectives suggest that a responsible AI ecosystem in Africa must be relational, situated, and reconstructive, addressing both the structural inequities of the present and the aspirational futures envisioned by African communities.

### Search strategy

To identify academic literature, searches were carried out on Google, Google Scholar and Scopus. Size, scope of these databases was considered when selecting literature to include in our analysis. To identify non-academic literature review key organisational websites and Google were used. Keywords used for the search included *‘AI’*, *‘Responsible AI in Africa’* AND, OR *‘Ethics, AI AND Africa’* OR *‘Trustworthy, Just, AI, Africa’* covering the period 2010 to 2025. Only academic works published in English by African scholars in Africa or beyond were included in the review. At the same time, only blogs and reports that were considered relevant in terms of focus on responsible AI in Africa were selected.

### Screening process

All academic works were initially screened for duplicates. Then they were assessed based on relevance and authorship by two researchers who independently read the paper’s title, abstract and authors list. Following this initial assessment, full-text retrieval and screening was conducted. Further works that could not be retrieved were removed. Subsequently, three researchers had discussions to harmonise individual assessments. The same process was followed for the non-academic works where blogs and reports identified were assessed for relevance to the topic. In the end, 63 works were selected for review: 19 peer reviewed Journal publications, 13 Blogs, 10 Book Chapters, 8 Preprints, 5 Reports, 4 Books, 3 Conference papers and 1 Website. See Table 1 for the full list of the reviewed works. Key themes and points of interests were then extracted.

**Table 1:** List of reviewed works

No	Selected works	Type
1	Adamu, M.S. (2023) No More “Solutionism” or “Saviourism” in Futuring African HCI: A Manyfesto. ACM Trans. Comput.-Hum. Interact., 30(2), p. 21:1–21:42	Journal
2	Ade-Ibijola, A. and Okonkwo, C. (2023) Artificial Intelligence in Africa: Emerging Challenges. In: Eke, D.O., Wakunuma, K. and Akintoye, S. (eds.) Responsible AI in Africa: Challenges and Opportunities. Cham: Springer International Publishing, pp. 101–117	Book Chapter
3	African Union (2024) Continental Artificial Intelligence Strategy | African Union. Ethiopia: AU	Report
4	Ahmed, S. (2021) A gender perspective on the use of artificial intelligence in Africa’s fintech industry: Case studies from South Africa, Kenya, Nigeria and Ghana. [Online] Research ICT Africa. Available from: https://researchictafrica.net/research/a-gender-perspective-on-the-use-of-artificial-intelligence-in-africas-fintech-industry-case-studies-from-south-africa-kenya-nigeria-and-ghana/ [Accessed 31/05/25]	Blog
5	Aiyegbusi, T. (2024) Pollicy. Reviews in Digital Humanities, [Online] Available from: https://reviewsindh.pubpub.org/pub/pollicy?readingCollection=7a79f2ab [Accessed 26/05/2025]	Website
6	Amugongo, L.M., Bidwell, N.J. and Corrigan, C.C. (2023) Invigorating Ubuntu Ethics in AI for healthcare: Enabling equitable care. In: Proceedings of the 2023 ACM Conference on Fairness, Accountability, and Transparency. FAccT’23. New York, NY, USA: Association for Computing Machinery, pp. 583–592	Conference paper
7	Awori, K. et al. (2016) Decolonising Technology Design. In: Proceedings of the First African Conference on Human Computer Interaction. AfriCHI’16. New York, NY, USA: Association for Computing Machinery, pp. 226–228	Conference paper
8	Benyera, E. (2021) The fourth industrial revolution and the recolonisation of Africa: The coloniality of data. Taylor & Francis	Book
9	Birhane, A. (2020) Algorithmic colonization of Africa. SCRIPTed, 17, p. 389	Journal
10	Borokini, F., Wakunuma, K. and Akintoye, S. (2023) The Use of Gendered Chatbots in Nigeria: Critical Perspectives. In: Eke, D.O., Wakunuma, K. and Akintoye, S. (eds.) Responsible AI in Africa: Challenges and Opportunities. Social and Cultural Studies of Robots and AI. Cham: Springer International Publishing, pp. 119–139	Book chapter
11	Butcher, N., Wilson-Strydom, M. and Baijnath, M. (2021) Artificial intelligence capacity in sub-Saharan Africa: Compendium report	Report
12	Eke, D. and Ogoh, G. (2022) Forgotten African AI narratives and the future of AI in Africa. The International Review of Information Ethics, 31(1)	Journal
13	Eke, D.O. et al. (2025a) African Perspectives of Trustworthy AI: An Introduction. In: Eke, D.O. et al. (eds.) Trustworthy AI: African Perspectives. Cham: Springer Nature Switzerland, pp. 1–17	Book chapter
14	Eke, D.O. et al. (eds.) (2025b) Trustworthy AI: African Perspectives. Cham: Springer Nature Switzerland	Book
15	Eke, D.O., Chintu, S.S. and Wakunuma, K. (2023) Towards Shaping the Future of Responsible AI in Africa. In: Responsible AI in Africa: Challenges and Opportunities. Springer International Publishing Cham, pp. 169–193	Book Chapter
16	Eke, D.O., Wakunuma, K. and Akintoye, S. (2023) Responsible AI in Africa: Challenges and Opportunities. Cham: Springer Nature	Book
17	Gaffley, M., Adams, R. and Shyllon, O. (2022) Artificial intelligence. African insight: A research summary of the ethical and human rights implications of AI in Africa. HSRC & Meta AI and Ethics Human Rights Research Project for Africa–Synthesis Report, [Online] Available from: https://africanaiethics.com/wp-content/uploads/2022/02/Artificial-Intelligence-African-Insight-Report.pdf [Accessed 25/05/2025]	Report
18	Gikunda, K. (2024). Harnessing Artificial Intelligence for Sustainable Agricultural Development in Africa: Opportunities, Challenges, and Impact. arXiv:2401.0617. 10.48550/arXiv.2401.06171	Preprint
19	Grancia, M.K. (2024) Decolonizing AI ethics in Africa’s healthcare: An ethical perspective. AI and Ethics, [Online] Available from: doi.org/10.1007/s43681-024-00650-z [Accessed 04/03/2025]	Journal
20	Gwagwa, A. et al. (2020) Artificial Intelligence (AI) Deployments in Africa: Benefits, Challenges and Policy Dimensions. The African Journal of Information and Communication, 26, pp. 1–28	Journal
21	Gwagwa, A. et al. (2021) Responsible artificial intelligence in Sub-Saharan Africa: landscape and general state of play. [Online] Available from: https://idl-bnc-idrc.dspacedirect.org/items/739b3b9a-6a8a-4b32-8db8-291cb621d2dd [Accessed 25/05/2025]	Blog
22	Gwagwa, A., Kazim, E. and Hilliard, A. (2022) The role of the African value of Ubuntu in global AI inclusion discourse: A normative ethics perspective. Patterns, 3(4), p. 100,462	Journal
23	Ibitoye, A.O. et al. (2025) Afrocentric Trustworthy Framework for Improved Artificial Intelligence Powered Health Management Tool for Africans. In: Eke, D.O. et al. (eds.) Trustworthy AI: African Perspectives. Cham: Springer Nature Switzerland, pp. 93–117	Book Chapter
24	Iyer, N. et al. (2021) Automated imperialism, expansionist dreams: exploring digital extractivism in Africa. Pollicy. June, [Online] Available from: https://scholar.google.com/scholar?cluster=7313927011706201036&hl=en&oi=scholarr [Accessed 21/05/2025]	Blog
25	Kalluri, P.R. et al. (2023). The Surveillance AI Pipeline. arXiv:2309.15084. 10.48550/arXiv.2309.15084	Preprint
26	Katushabe, I.M. (2023) Gendered aspects of Artificial Intelligence (AI) and its impact on Data protection for women and girls in Africa – WOUGNET. Available from: https://wougnet.org/gendered-aspects-of-artificial-intelligence-ai-and-its-impact-on-data-protection-for-women-and-girls-in-africa/ [Accessed 31/05/25]	Blog
27	Khan, M. and Ewuoso, C. (2024) Epistemic (in)justice, social identity and the Black Box problem in patient care. Medicine, Health Care, and Philosophy, 27(2), pp. 227–240	Journal
28	Kiemde, S.M.A. and Kora, A.D. (2021) Towards an ethics of AI in Africa: rule of education. AI and Ethics, pp. 1–6	Journal
29	Kuzmina, T., Podbiralina, G. and Baburina, O. (2024) Impact of artificial intelligence on sustainable development in African countries. E3S Web of Conferences, 583, p. 08005	Conference paper
30	Kwao, G.P., Kanubala, D.D. and Sonna, B. (2023) AI ethics education for future African leaders. AI Ethics in Higher Education: Insights from Africa and Beyond, p. 87	Book
31	Lee, J., Gamundani, A. and Stinckwich, S. (2021) Towards a Human Rights-Based Approach to Artificial Intelligence. [Online] United Nations University. Available from: https://unu.edu/macau/blog-post/towards-human-rights-based-approach-artificial-intelligence [Accessed 21/05/25]	Report
32	Lotzof, K. (2020) Your mobile phone is powered by precious metals and minerals. [Online] Natural History Museum. Available from: https://www.nhm.ac.uk/discover/your-mobile-phone-is-powered-by-precious-metals-and-minerals.html [Accessed 29/05/25]	Blog
33	Mathur, V. et al. (2023) AI, Climate Adaptation, and Epistemic Injustice. [Online] Transitions Research. Available from: https://transitionsresearch.org/ai-climate-adaptation-and-epistemic-injustice/ [Accessed 04/03/25]	Blog
34	Mienye, I.D., Sun, Y. and Ileberi, E. (2024) Artificial intelligence and sustainable development in Africa: A comprehensive review. Machine Learning with Applications, 18, p. 100,591	Journal
35	Muzanenhamo, P. and Power, S.B. (2024) ChatGPT and accounting in African contexts: Amplifying epistemic injustice. Critical Perspectives on Accounting, 99, p. 102,735	Journal
36	Ndaka, A. et al. (2024) Toward response-able AI: A decolonial perspective to AI-enabled accounting systems in Africa. Critical Perspectives on Accounting, 99, p. 102,736	Journal
37	Ndiaye, S.M. (2025a) A Roadmap for AI Sovereignty in Africa. [Online] Apolitical. Available from: https://apolitical.co/solution-articles/en/a-roadmap-for-ai-sovereignty-in-africa [Accessed 25/05/25]	Blog
38	Ndiaye, S.M. (2025b) Building Trustworthiness as a Requirement for AI in Africa: Challenges, Stakeholders and Perspectives. In: Eke, D.O. et al. (eds.) Trustworthy AI: African Perspectives. Cham: Springer Nature Switzerland, pp. 41–67	Journal
39	Nkwo, M.S. and Adamu, M. (2025) Trust Me, I Am an Intelligent and Autonomous System: Trustworthy AI in Africa as Distributed Concern. In: Eke, D.O. et al. (eds.) Trustworthy AI: African Perspectives. Cham: Springer Nature Switzerland, pp. 69–92	Book Chapter
40	Nothias, T. (2025) An intellectual history of digital colonialism. Journal of Communication, p. jqaf003	Journal
41	OECD (2024) Artificial intelligence in science: challenges, opportunities and the future of research. Paris: Fondation Ipsen BookLab	Report
42	Ojadi, J. et al. (2024) AI-Enabled Smart Grid Systems for Energy Efficiency and Carbon Footprint Reduction in Urban Energy Networks. International Journal of Multidisciplinary Research and Growth Evaluation, 05, pp. 1549–1566	Journal
43	Ojika, D., Strayer, J. and Kaul, G. (2021). Towards Sustainable Energy-Efficient Data Centers in Africa. arXiv:2109.04067. 10.48550/arXiv.2109.04067	Preprint
44	Okolo, C.T., Aruleba, K. and Obaido, G. (2023) Responsible AI in Africa—Challenges and Opportunities. In: Eke, D.O., Wakunuma, K. and Akintoye, S. (eds.) Responsible AI in Africa: Challenges and Opportunities. Cham: Springer International Publishing, pp. 35–64	Book Chapter
45	Okorie, C. and Marivate, V. (2024) How African NLP Experts Are Navigating the Challenges of Copyright, Innovation, and Access. [Online] Carnegie Endowment for International Peace. Available from: https://carnegieendowment.org/research/2024/04/how-african-nlp-experts-are-navigating-the-challenges-of-copyright-innovation-and-access?lang=en [Accessed 27/03/25]	Blog
46	Orero, L.O. (2024) Cracking the Gender Equality Code in African AI through the Datasphere. [Online] The Datasphere Initiative. Available from: https://www.thedatasphere.org/news/cracking-the-gender-equality-code-in-african-ai-through-the-datasphere/ [Accessed 20/05/25]	Blog
47	Orife, I. et al. (2020). Masakhane – Machine Translation For Africa. arXiv:2003.11529. 10.48550/arXiv.2003.11529	Preprint
48	Ormond, E. (2023). Governance of AI Ethics: Perspective from the Global South (Africa). 10.2139/ssrn.4368020	Preprint
49	Owoyemi, A. et al. (2020) Artificial Intelligence for Healthcare in Africa. Frontiers in Digital Health, 2, p. 6	Journal
50	Owoyemi, A. et al. (2025) Trustworthy AI in Healthcare: Exploring Ethics in Digital Health Technologies in Nigeria. In: Eke, D.O. et al. (eds.) Trustworthy AI: African Perspectives. Cham: Springer Nature Switzerland, pp. 193–206	Book Chapter
51	Pasipamire, N. and Muroyiwa, A. (2024) Navigating algorithm bias in AI: ensuring fairness and trust in Africa. Frontiers in Research Metrics and Analytics, 9, p. 1,486,600	Journal
52	Pell, R. et al. (2021) Towards sustainable extraction of technology materials through integrated approaches. Nature Reviews Earth & Environment, 2(10), pp. 665–679	Journal
53	Ravindran, S. (2023) AI often mangles African languages. Local scientists and volunteers are taking it back to school | Science | AAAS. Available from: https://www.science.org/content/article/ai-often-mangles-african-languages-local-scientists-and-volunteers-are-taking-it-back [Accessed 20/05/25]	Blog
54	Ruttkamp-Bloem, E. (2023) Epistemic Just and Dynamic AI Ethics in Africa. In: Eke, D.O., Wakunuma, K. and Akintoye, S. (eds.) Responsible AI in Africa: Challenges and Opportunities. Cham: Springer International Publishing, pp. 13–34	Book Chapter
55	Salami, A.O. (2024) Artificial intelligence, digital colonialism, and the implications for Africa’s future development. Data & Policy, 6, p. e67	Journal
56	Sam, A.K. and Olbrich, P. (2023) The Need for AI Ethics in Higher Education. In: Corrigan, C.C. et al. (eds.) AI Ethics in Higher Education: Insights from Africa and Beyond. Cham: Springer International Publishing, pp. 3–10	Journal
57	Sey, A. and Ahmed, S. (2020) An African perspective on gender and artificial intelligence needs African data and research. [Online] Research ICT Africa. Available from: https://researchictafrica.net/research/an-african-perspective-on-gender-and-artificial-intelligence-needs-african-data-and-research/ [Accessed 31/05/25]	Blog
58	Siminyu, K. et al. (2020). AI4D – African Language Dataset Challenge. arXiv:2007.11865. 10.48550/arXiv.2007.11865	Preprint
59	Varshney, K.R. (2024). Decolonial AI Alignment: Openness, Viśe\d{s}a-Dharma, and Including Excluded Knowledges. arXiv:2309.05030. 10.48550/arXiv.2309.05030	Preprint
60	Wakunuma, K. et al. (2025) Decoloniality as an Essential Trustworthy AI Requirement. In: Eke, D.O. et al. (eds.) Trustworthy AI: African Perspectives. Cham: Springer Nature Switzerland, pp. 255–276	Book Chapter
61	Wheeler, A. (2018) What Raw Materials Are Used to Make Hardware in Computing Devices?. [Online] Engineering.com. Available from: https://www.engineering.com/what-raw-materials-are-used-to-make-hardware-in-computing-devices/ [Accessed 29/05/25]	Blog
62	Yeboah, K. (2020) Artificial Intelligence (AI) and Inclusive Innovation: Examining Contemporary AI Initiatives in sub-Saharan Africa. [Online] Available from: https://era.library.ualberta.ca/items/739df46f-e639-4e1e-842e-bf0fc4bb00f0 [Accessed 25/05/2025]	Blog
63	Yilma, K. (2024). Ethics of AI in Africa: Interrogating the Role of Ubuntu and AI Governance Initiatives. Ethics and Information Technology, Vol. 27, No. 2 (2025), Available at SSRN: https://ssrn.com/abstract=4910877	Preprint

### Data extraction and analysis

Data extraction was conducted using a shared spreadsheet, with fields capturing bibliographic details, publication type (academic or grey literature), geographic and institutional focus, and a free-text summary of each work's central claim or concern regarding responsible AI in Africa. The spreadsheet was piloted by three researchers, who independently extracted the first two works; inconsistencies in what counted as a relevant "concern" were discussed and resolved before the remaining works were coded.

**Fig. 3:**
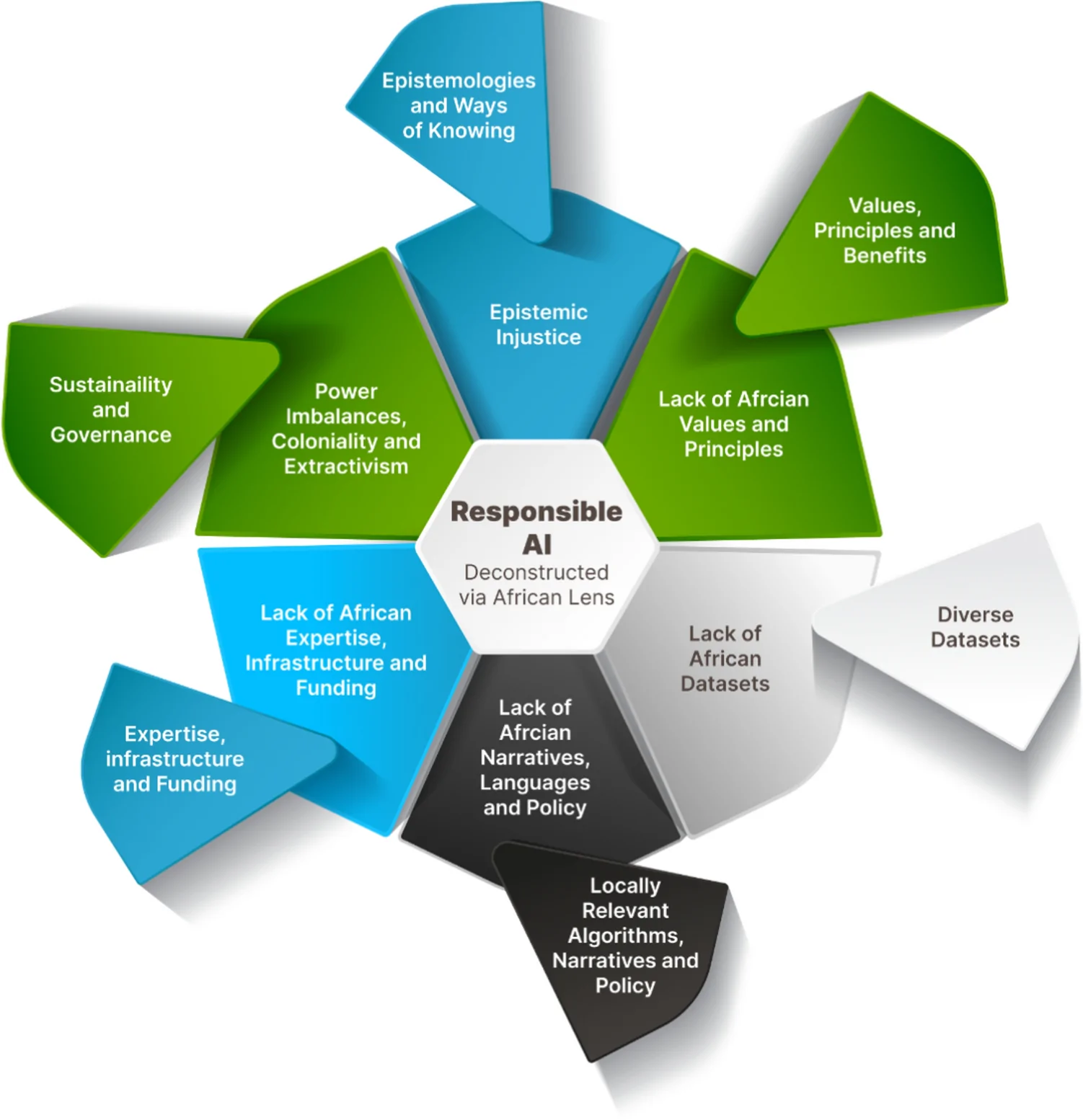
Responsible AI- deconstructed via African Lens: This figure mirrors Fig. 1's six dimensions exactly, but relabels each as the specific critique African scholars raise against it. The one-to-one pairing with Fig. 1 is deliberate: each wedge shows what "deconstructing" that same global dimension reveals

Theme identification followed an inductive thematic analysis approach [23, 24]: each work's central concern was first open-coded in the researchers' own words; codes were then grouped by conceptual similarity into identified categories; and these categories were iteratively reviewed and merged by the research team until they converged into the six categories reported in Sect. 5 and Fig. 3. Disagreements over category boundaries were resolved through group discussion rather than by majority vote.

Works were not assigned numerical weights, and no work's inclusion in a category depended on a formal scoring procedure. Instead, a small number of works recur across several categories rather than being confined to one. These recurring works supplied much of the conceptual vocabulary, epistemic injustice, algorithmic colonisation, decoloniality, against which the remaining, more narrowly scoped works were interpreted, and are accordingly more influential in shaping the categories than works cited once in support of a single point. Table 1 summarises the composition of the reviewed corpus by publication type and category, distinguishing academic from grey-literature sources, to give a transparent, auditable account of how the 63 reviewed works map onto the six thematic categories.

## Findings (deconstruction of afrocentric responsible AI)

Our findings show that African scholars have actively been engaged in the deconstruction of global discourses on responsible AI. The available body of work highlights how global AI and responsible AI discourses, often produced by institutions and scholars in the Global North, fail to account for the socio-historical, economic, and epistemological contexts of African societies [9, 10, 13, 25]. African scholars are increasingly critiquing the universalist tendencies in AI and responsible AI principles, arguing that they often replicate colonial patterns of knowledge production and technological extractivism [5, 26]. Through this epistemic deconstruction, they expose the limitations of importing non-African models of fairness, personhood, accountability, and transparency into African settings without addressing underlying structural inequalities or engaging with local value systems. This critique is not merely oppositional; rather, it creates a space for constructive efforts that centre Afrocentric, relational, and community-informed approaches to ethical AI. In sections below, we detail these deconstruction narratives as they are presented in literature (see Fig. 2 for graphic representation).

### Lack of African epistemologies/epistemic injustice

The first level of deconstruction found in literature is in the epistemic foundations of global responsible AI [27–31]. These scholars highlight the epistemic asymmetries that characterise the global AI and responsible AI discourse, where African voices and scholarship are often underrepresented or framed within deficit narratives. This is conceptualised as "epistemic injustice" [15], which manifestsin the neglect of African perspectives in global discussions as well as in the importation of AI ethics frameworks that do not adequately capture African epistemologies; ways of knowing, knowledge systems, and meanings rooted in African cultures, philosophies, histories, and lived experiences [9, 32]. These are critical epistemic elements that help AI to address local priorities such as digital and data sovereignty, communal or communitarian values, or socio-economic justice. Mathur et al., [33] identified epistemic injustice in the context of climate change which can limit the understanding of complex environmental issues and hinder the development of comprehensive and contextually relevant climate solutions. Identification of epistemic injustice here challenges or attempts to deconstruct the global perspective of responsible AI in terms of: who defines what counts as "responsible"?, whose knowledge is legitimised, and whose is excluded? [10]. This is critical because global AI ethics frameworks often assume a universal ethics whilst drawing from European and American liberal traditions, prioritise values such as individual rights over collective or relational values and emphasise technical fixes while neglecting historical injustices, coloniality, or extractive data economies [5].

Africa is characterised by varied values, beliefs, systems of knowing, and unique experiences which inform how the people produce and represent knowledge. When it comes to the African epistemologies, various studies have critiqued the lack of epistemic justice (that is, the exclusion and/or systematic misrepresentation of indigenous values, knowledge and experiences), which have pervaded the AI ethics and governance ecosystem. For instance, Ruttkamp-Bleom [28] considered the absence of an indigenous system-like structure that is dynamic, actionable, and could drive the actualisation of the goals of responsible AI ethics and AI policymaking among African societies. This becomes imperative because the fast-changing nature of emerging technologies such as AI requires an inter-, trans, and multi-disciplinary research approach that will consider the impact of context and culture on the successful adoption of AI technologies in general and on trust in AI technology and openness to AI regulation [ibid]. These would engender dynamic and epistemic applications of AI ethical regulations across multiple domains and cultures, which are critical in enhancing conversations on ethics. In their commentary on the epistemic injustice, to the development of accounting knowledge and practice in, and on, Africa, Muzanenhamo and Power [30] highlight how ChatGPT perpetuate epistemic injustice through anonymisation and lack of transparency and traceability of data sources by alienating accounting knowledge producers, especially from Africa, from the products of their labor in contrast to the West whose epistemologies were employed to build the Large Language Model (LLM). The authors argue that this development actively amplifies the power imbalance that exists in the global knowledge production and dissemination system thereby marginalising unique epistemologies of accountants from Africa [ibid]. The authors also suggest, among other things, the adoption of regulations which ensure that accounting knowledge reproduced by LLMs such as ChatGPT is transparent and traceable to the original producers/sources, which helps to check epistemic injustice and prevent existing alienation of knowledge producers from their works [30].

### Lack of African values

As part of the discussion on epistemic injustice, many scholars have identified the lack of African values, principles and contexts in the global discussion on responsible AI [27, 32, 34–39]. These scholars identify the total absence of or marginalisation of African values such as *Ubuntu* (i.e., the emphasis on humanity and interconnectedness)*, Ujamaa* (i.e., socialist ideology on cooperative economics)*, Umunna* (the focus on social and family systems), communality or spirituality, in global AI ethics frameworks. They expose that these frameworks and values are not neutral, universal, or value-free, as they often claim to be. These scholarly works show that the global responsible AI discourses often present principles like fairness, transparency, accountability, and privacy as if they are universal values. But, these are often derived from Western liberal, individualistic and largely rights based.

Responsible and trustworthy AI and AI ethics often draw from legal, philosophical, and technical traditions rooted in cultural contexts. By identifying the lack of African values these scholars unpack the boundaries of the concept responsible AI and highlight the epistemically narrowness of the discourse. These framings also point out that the displacement of African imaginaries and values are not accidental but more so a result of structural power imbalances in who produces AI knowledge, who sets ethical standards, and who gets consulted. As noted by Pasquinelli [40] in the social genealogy of AI, “power was no longer concerned with the discipline of the individuals but the control of *individuals*, that is of the fragments of an extended and deconstructed body” (p.242). The deconstruction here draws attention to these power imbalances.

### Lack of African datasets

As AI models and technologies advance and more sophisticated AI tools are built, the digital data divide between low-resource languages and others will likely get even bigger. This is because the common prerequisite of these advanced systems is the existence of large amounts of digital data. However, studies have shown that a lack of African datasets and/or inaccessibility of relevant African data on the Internet leads to the systematic exclusion of relevant African datasets in developing AI systems [27, 41–43]. As a result, many existing machine learning models struggle to understand and properly interact with various kinds of user prompts and queries. In their studies, Okolo et al. [39] analysed the factors that impact the adoption and successful implementation of AI technologies in Africa. The result from this study reveals that there is little work regarding big digital datasets, among other factors, which could have helped to understand the nuances and sociotechnical implications of AI development in Africa compared to the West and China. Combined with other factors such as a lack of homegrown AI policies, the study also opines that a lack of relevant African datasets fosters continued exploitation of the continent for profit [ibid], a concept that has also been referred to as digital or data colonialism [44, 45].

Moreover, there is a lack of relevant datasets for use in AI models across various application domains. For example, studies on the use of AI in health and medicine in Africa reveal that, one of the main challenges, among other challenges, is the lack of large clinical datasets for training AI models [46, 47]. While annotation of datasets by medical professionals is usually costly and time-consuming, the low level of digitisation and electronic medical record use on the continent leads to the scarcity of indigenous and quality health datasets required for training AI models [46]. While advocating for the widespread utilisation of electronic medical records and the maintenance of large-scale medical databases, the paper [ibid] suggested the acceleration of the ongoing improvement in Africa's infrastructure, especially electricity and Internet penetration, will catalyse data generation and analysis needed for advanced automation of processes involved in patient care.

### Lack of locally developed or culturally relevant algorithms (in other words, lack of African narratives)

Another level of deconstruction found in the literature is lack of locally and culturally relevant algorithms in the existing AI systems which stifles indigenous values while promoting Western perspectives, and effectively leaving the continent dependent on Western ideologies, software, and infrastructure[13, 32, 35, 48]. Birhane [13] examined how Western tech monopolies, with their desire to dominate, control, and influence social, political, and cultural discourse, share common characteristics with traditional colonialism. The paper drew distinct examples of how algorithmic colonisation has been foisted from various parts of the continent and outlined a vision of AI that is rooted in local community needs and interests. The author argues that the vision will be invaluable as it will engender the design of AI products that are locally appropriate and meet the needs of African users, instead of current AI products, which are usually unfit for African needs [ibid]. The near absence of AI policies that would regulate the proliferation of AI technologies on the African continent gained the attention of Gwagwa et al. [41], who highlighted some of the core AI opportunities, challenges, and policy dimensions requiring the attention of African policymakers.

Drawing on examples from financial inclusion to combating cultural and linguistic marginalisation, the reviewed literature highlights that stakeholders responsible for creating policies that will aid the adoption of AI innovations designed to support various areas of African society, economy, and government must be mindful of the following key dimensions: gender equity, cultural and linguistic diversity, and labour market shifts. Such AI policies must be extensively informed by the distinctive characteristics of the African continent [37]. While Eke et al. [32] argued for the clarification of the importance of responsible AI that considers African contexts, interests, values, fears, hopes and aspirations, Kiemde and Kora, [49] believed that it is difficult to implement proposed solutions for the development of ethical and responsible AI in Africa without the integration of ethical concepts in the training of actors. Both studies reviewed current works on the ethics of AI in Africa. The authors recommended distinct approaches to embed African value systems and principles in Afrocentric AI systems via philosophies such as *Ubuntu, Ujamaa, Umunna* and others in the framing of a localised AI for Africa [32] and the introduction of ethics courses in academic training and capacity building of AI development actors [48].

Furthermore, the underlying global narrative of responsible AI is also a worrying linguistic gap and inequity, where African languages are either entirely missing or included in distorted and tokenistic ways. This not only marginalises the vast population of Africans from meaningful participation in AI design, development and use but also entrenches epistemic injustice by erasing culturally grounded ways of knowing and communicating. For example, the Hausa language ‘‘is the second most spoken African language, with an estimated 60 million to 80 million speakers, and it is just one of more than 2000 African languages that are mostly absent from AI research and products’’ [50]. The impacts of this linguistic marginalisation are far-reaching, from the fairness and interpretability of AI algorithms to the equitable distribution of AI's benefits. The available global training data for AI lacks representation from African languages. Therefore, AI models inevitably perform poorly in African linguistic contexts which can lead to misdiagnosis in healthcare, inaccurate legal advice, or biased outputs. Without the ability to engage with AI in their native languages, African communities are denied true agency in shaping AI's trajectory, hindering their capacity to advocate for responsible AI development that genuinely serves their unique societal needs, contexts and aspirations. This can amount to what can be referred to as ‘exclusion-by-design’. Highlighting this in a keynote speech at the AI for Good Global Summit, Pelonomi Moila, CEO of Lelapa AI noted that ‘‘if you don’t speak one of the major European languages, you cannot access digital products and services which are not multilingual—from chatbots to voice instructions or government services’’.^1^ To bridge this gap, some initiatives have been established in Africa by several scholars [42, 51, 52], who have established communities (such as Deep Learning Indaba^2^) that seek to strengthen the African AI ecosystem by building capacity and supporting the development of AI systems that work for the continent. In this paper, we conclude that without the integration of languages that can improve access and shared benefits, we cannot achieve a truly global view of responsible AI.

### Lack of African expertise and funding

Another level of deconstruction we discovered in the literature involves the challenges associated with limited Indigenous AI expertise, infrastructure, and severe funding gaps in the research and development of AI technologies [7, 27, 53]. Following their inquiry to understand why Africa’s AI narratives are often missing in the global AI narrative, Eke and Ogoh, [27] identified the prejudices behind not valuing the expertise of Africans within the tech community, or African socio-cultural, ethical and political contexts and narratives in global responsible AI debate as one of the key challenges. In another instance [54] highlighted the lack of capacity for AI in African contexts. Thus, Eke and Ogoh [27] suggested ways to create a more responsible and culturally sensitive AI for Africa, which considers broader indigenous principles and values that are necessary for the advancement of AI expertise and infrastructure on the continent [27]. Also, among other results, [55] uncovered that improvement in Internet infrastructure, stable electricity, and developing digital skills and literacy are key to ensuring AI inclusivity on the African continent. Moreover, while Ade-Ibijola, and Okonkwo, [53] highlighted scarcity of AI-ready skilled workers for various application areas as one of the major issues facing the adoption of AI on the continent, Gwagwa et al., [7] emphasised the need for Africans to participate in the development of AI technologies that consider the socio-economic and infrastructural realities of the continent. The study suggested four core areas of focus: innovation, capacity building, policy, and infrastructure, which could be the turning point in the development of a responsible AI ecosystem within which researchers, innovators, and innovations can flourish [7]. In an interview in February 2025, Dr Seydina M. Ndiaye; a member of the UN Secretary-General’s High-Level Advisory Body on Artificial Intelligence identified capacity building, developing AI expertise within the continent and increased investment in AI by Africa’s public and private sectors as two of the five key areas put forward in the Pan-African AI strategy [56, 57]. A report on the roadmap to African sovereignty presented some recommendations and argued that encouraging public and private investment and using global partnerships to enhance AI capabilities across Africa are critical to realizing the benefits of AI and mitigating associated risks in deployment [56]. In addition, a 2023 Organisation for Economic Co-operation and Development (OECD) report highlighted the importance of accelerating the productivity of research and uptake of AI in science [58]. This, they believe, would make it more economically and socially valuable, thereby helping OECD countries to grow, innovate, and address global challenges, from climate change to new contagions, and actualise its full potential. Furthermore, we argue that insufficient and sometimes the absence of necessary AI infrastructure, limited funding, and prevailing socio-cultural barriers are significantly contributing to the paucity of needed datasets across various application domains. Therefore, our current study agrees with these existing studies, which suggest that addressing the skills, infrastructural, and funding gaps would help the continent to craft AI solutions that are truly indigenous and serve the continent’s priority needs while helping its economies realise the United Nations Sustainable Development Goals (SDGs).

### Increasing power imbalances and coloniality in the AI ecosystem

In Africa, the common geo-political narratives about AI centre the theme of socio-economic development and fourth industrial revolution (4IR): AI as an instrument for rescuing failed emerging technologies. In Human–Computer Interaction (HCI) research for example, [59] has shown how Western-driven techno-solutionism narratives have relegated the African technology innovation ecosystem around tropes “lacks” and “gaps”, and ultimately, modern Western imaginaries are sought to mitigate those institutional predicaments. However, others have questioned such premises, arguing instead that as long as Western modernity has furnished the diversity of coloniality and prosperity, emerging technologies as a Western-led tool of power will only amplify those historical forces towards the unequal futures [60]. As research and innovation in African HCI has demonstrated, the tropes of appropriation, adaptation, and localisation of Western technologies are implicated by Western design aesthetics and methods that have failed to drive technology development. With the misalignment of AI as a development optic or an instrument of the 4IR, there is an apparent lack of trust in the wider AI pipeline, particularly as they relate to whose worldview, whose knowledge and whose labour shape the future of AI [61].

Scholars are also drawing attention to the inherent coloniality in the AI ecosystem [5, 62, 63]. As Wakunuma et al., [5] put it: ‘‘there is sufficient evidence from literature to show that AI systems often perpetuate and exacerbate historical patterns of domination, exploitation, hierarchies of power, and inequality/marginalising that are reminiscent of colonial practices’’. An aspect of responsible AI that has not received the same spotlight as machine learning is computing hardware, which provides processing, compute and storage for machine learning and computational resources. In their review, Pell et al., [64] describe the process of technology materials going from mining locations around the world to being embedded in low-carbon technology. Such raw materials, which are required for electronics and computer hardware, include copper, cobalt, tellurium, lithium, cobalt, manganese, ophiolite and tungsten. These raw materials and critical minerals are sourced from locations around the world, with a big percentage coming from the African continent in locations such as the Democratic Republic of Congo (DRC), Gabon, Ghana, Guinea, Madagascar, Morocco, Mozambique, South Africa, Zambia, and Zimbabwe, among others [64, 65]. For example, the largest deposits of cobalt (as high as 70% from a single source) required for batteries, and tantalum which are used for capacitors, are sourced from the DRC [65, 66]. This means that Africa is now one of the AI resources frontiers; from raw materials mining, unprecedented data generation to the exploitative use of cheap labour for data labelling [67]. With the increasing demand for raw materials, the emphasis here is that responsible AI cannot be detached from global and climate justice. The exploitation of African environments and communities for powering AI infrastructure is increasingly being critiqued as a form of colonialism. Criticisms here are rooted in a commitment to ecological relationality, and a demand for resource and data justice because of the systemic extractivism. In essence, the moral absolutism, material extractivism, cultural essentialism, and digital surveillance associated with the AI lifecycle are increasingly being highlighted [13, 60].

## Critical discussion—reframing the concept of responsible AI

The findings mapped above can be read through the three interlocking dimensions of coloniality set out in Sect. 3. The lack of African epistemologies and the epistemic injustice this produces are expressions of the coloniality of knowledge: the treatment of Eurocentric AI ethics vocabularies as universal rather than as one regional tradition among many. The absence of African datasets, infrastructure, expertise and funding reflect the coloniality of power: the concentration of the material and institutional resources that shape AI in the Global North. And the marginalisation of African values and gendered harms, together with the framing of African users as recipients rather than authors of AI systems, point to the coloniality of being: the persistent classification of African subjects as objects of AI design rather than agents of it. However, identifying coloniality in this way across the findings is not an end in itself. Consistent with the decolonial theoretical lens, this critical and deconstructive phase is a precursor to a constructive one: a reframing and reimagining of what responsible AI can mean when it is not assumed to have a single epistemic centre.

Critical to the construction process is the acknowledgement of the diversity of cultures, realities, contexts and jurisdictional provisions in Africa. African culture is not monolithic. But as Eke et al., [10, 68] pointed out, the majority of African societies share common colonial heritage and influence that shapes our diverse realities and aspirations. This underlines the interpretation of suggested construction efforts in literature. To address the colonial legacies inherent in the current AI lifecycle, which is often neglected in global responsible AI discourse, Wakunuma et al., suggest the inclusion of decoloniality as a necessary requirement for trustworthy AI. According to these scholars, ''decoloniality ensures that AI systems are designed with a deep understanding of the cultural, social, and economic realities of the communities they serve, which is essential for trustworthiness'' [5]. A new meaning of responsible AI in Africa should therefore include decoloniality as a critical element. To counter apparent epistemic injustice in AI systems, Ruttkamp-Bleom [28] suggested an epistemic just and dynamic AI ethics framework for Africa. For her, the prioritisation of African epistemologies and a dynamic ethical worldview in AI regulations and governance models are the foundations for responsible AI in Africa. This includes critical education of future generations on the need to embed indigenous values into AI design and development [28].

There are also emerging feminist approaches to responsible AI in Africa that reflect this theoretical lens. These are taking shape through grassroots community organising, activism and interdisciplinary collaborations. These approaches, in the form of intersectional justice (e.g. Pollicy in Uganda [69]), community-led data sovereignty (Feminist AI Research [FAIR] Lab Africa) and resistance to algorithmic patriarchy [70] are critical to a new meaning of responsible AI in Africa. Intersectionality here is a design principle that should account for intersecting oppressions of gender, race, class and disability. They emphasise data sovereignty and digital self-determination, particularly for women and informal workers. In many parts of Africa, feminist AI scholars are resisting gendered AI harms [70–74].

Contrary to the rights-based [75] and risk-based [76] approaches that characterise global narratives of responsible AI, African scholars are increasingly advocating for a value-based approach [32, 35, 77]. This is because traditional African principles, philosophies, and indigenous knowledge, such as Ubuntu, relationality, connectedness, and communal understanding, consensus building, drive the way people perceive and utilise emerging technologies across diverse application domains [35, 78–80]. As a result, there is a need for careful and contextual approaches to aligning AI systems to the traditional values, perspectives, and idiosyncrasies of the people. Value-based design approaches to building responsible AI systems, which are constructed in line with these indigenous African principles and philosophies, come in handy in these circumstances. Elements of this are already contained in the first continental strategy related to AI in Africa where AU member States are seeking to collaboratively establish unified legal systems that clearly define AI ethics, offering protection and binding obligations across the continent [81].

For instance, in comparison to the common values that represent design and technology in the West, the results from Afrocentric participatory design research conducted in Namibia highlights the need for AI technology developers to be sensitive to the specificities in African communities with diverse cultural and social values that inform their way of knowing [82]. The study, which was conducted amongst the Herero people in Namibia, revealed the difference in how indigenous knowledge is transmitted among the people through communal interaction and traditional teaching approaches [82], and these have implications for enhancing social interaction and effective user experience with AI tools. Also, Ibitoye et al. [79] and Amugongo et al. [78] investigated how Afrocentric AI tools could be designed to promote trust, transparency, and strengthen collaborative healthcare services (hallmark of the spirit of Ubuntu). While Ibitoye et al. [79] proposed and conceptualised a framework integrating responsible AI principles with Africa's social values in tailoring local healthcare complexities to enhance user trust and acceptance, initial results from Amugongo et al. [78] shows how relationality as represented in the concept of Ubuntu can bring about equity and equality as relevant responsible AI principles. These results are significant and reveal locally appropriate strategies for translating responsible AI principles into understandable design guidelines for critical health-based AI systems that will address localised concerns of health data privacy and safety, fairness, and accountability by design.

There is also a growing African scholarship on sustainable AI focused not just on reducing emissions, but also on reimagining AI ecosystems that are regenerative, locally grounded and socially accountable, an orientation that is itself a form of resistance to the coloniality of power, which concerns not only who sets ethical standards but who controls the material infrastructure, minerals, energy, data centres, on which AI depends. There is an emphasis on resource sovereignty (including as regards data). Artificial Intelligence for Development (AI4D) supports projects using low-resource models and edge computing to avoid heavy data infrastructure [83]. Ojadi et al., [83] called for decentralised, low-carbon AI solutions that prioritise human and environmental wellbeing. Whilst emphasising the need to pursue green or sustainable AI, which is more environmentally friendly and inclusive than conventional AI, Mienye et al., [84] described AI solutions that foster eco-friendly practices in other fields (green-by AI), strategies for designing energy-efficient machine learning algorithms and models (green-in AI), and tools for accurately measuring and optimising energy consumption. Among other things, scholars are exploring emerging trends in sustainable AI designs which help to develop AI devices that can harvest energy from the surrounding environment to power AI systems from ambient light, vibrations, and heat [85, 86]. This makes AI systems more self-sufficient, reducing the need for external power sources, and shaping a more sustainable and environmentally conscious future.

To ensure that there is a focus on the environmental impact of mining natural resources that shape the AI infrastructure as well as the huge carbon footprints of AI data centres in Africa, scholars are emphasising that there should be a focus on ecological/environmental sustainability [87]. Studies highlight the efforts being made to institutionalise responsible AI practices in the research, design, and deployment of AI systems that support organisational processes and products, and which helps to foster greater ecological integrity and social justice, particularly in emerging economies across Africa. In addition to engaging with innovations and Afrocentric frameworks and methodologies to AI design which align with a sustainable and automated future [79], enhanced research collaboration between Western AI companies and indigenous AI designers and researchers in Africa will promote sustainability by engendering reverse mentoring which ensures the development of a deeper understanding of the appropriate adjustments to AI process models and algorithms.

**Fig. 4:**
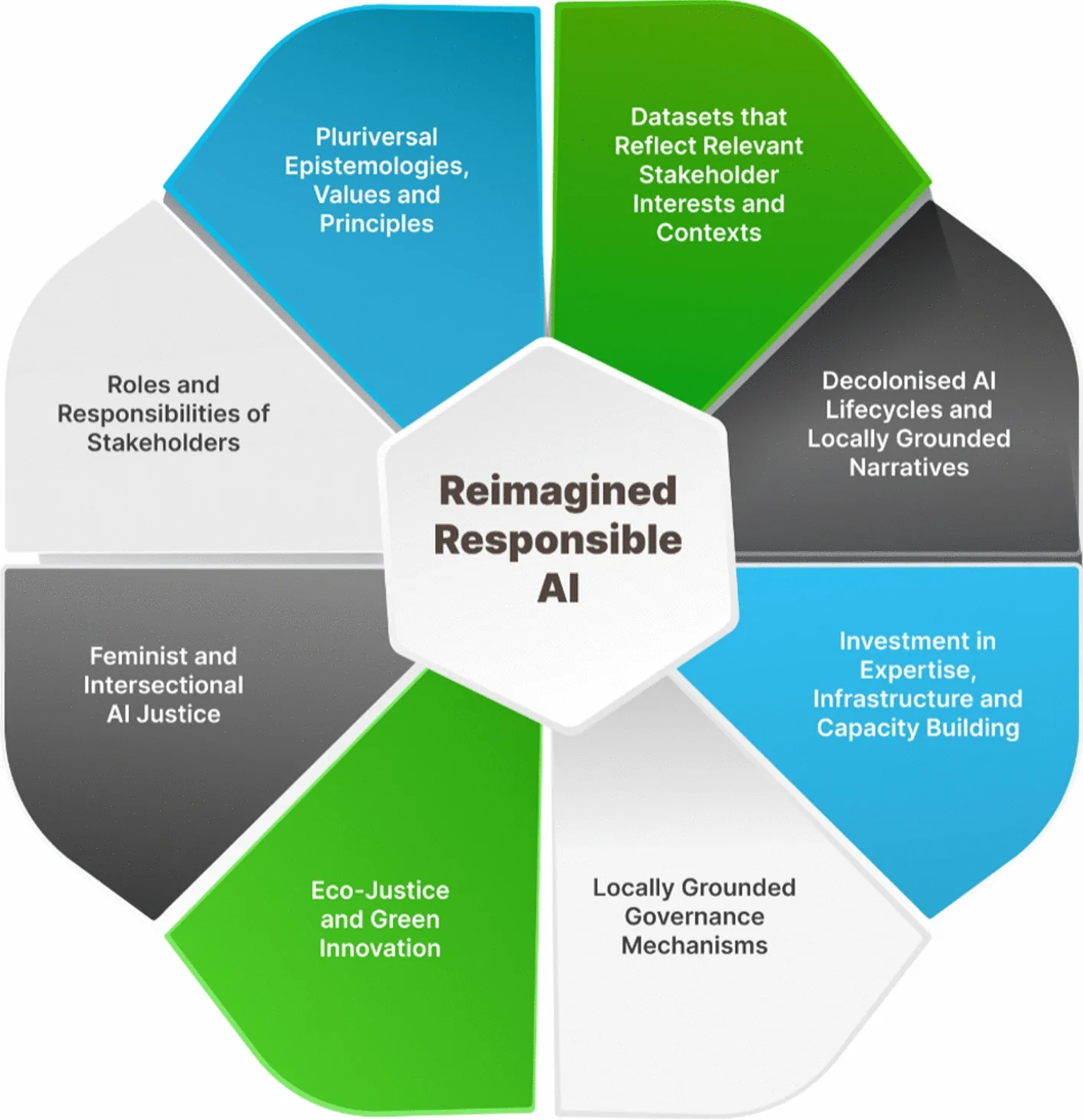
Reimagined responsible AI: Eight wedges showing the constructive, decolonial counterpart to each critique in Fig. 3, epistemologies and values, datasets, AI lifecycle and narratives, expertise and infrastructure, governance, and environmental justice, plus Feminist and Intersectional AI Justice, one further form decoloniality takes in response to the coloniality of being, alongside Ubuntu and other relational value systems

We must provide a critical caution in this move from critique to construction, which is the risk of merely reversing the hierarchy we seek to unsettle. Our bid to highlight African epistemologies ought not to amount to establishing a new epistemic "centre" in place of a displaced Western universalism. Such a reversal would replicate the very logics of centralisation and exclusion that our analysis exposes. In line with Mignolo's caution that detachment from a colonial matrix of power should not simply install a rival universal in its place [14], and Ndlovu-Gatsheni's insistence that deprovincialising Africa must go together with provincialising Europe rather than replacing one centre with another [88], our call for decoloniality is not an attempt to essentialise "African values" or present them as a unified normative foundation. African contexts are plural, historically layered, and internally diverse. This constructive turn we advocate for should therefore be understood as pluriversal rather than substitutive: not the replacement of one universal with another, but the opening of responsible AI to a multiplicity of co-existing epistemologies and value systems. In this sense, decolonial reconstruction is not about suggesting Africa as a new moral authority but about displacing the very assumption that responsible AI must have a single epistemic centre at all. Figure 4 presents a graphical representation of the reimagined responsible AI model that is pluriversal.

## Conclusion and future directions

This paper began by asking whose idea of responsibility is embedded in AI systems. The decolonial theory underlying our findings suggests that this question has, until now, been answered largely by default: responsibility for defining, governing and adjudicating "responsible AI" has been assumed by institutions, scholars and standard setters in the Global North. This default allocation of responsibility is not neutral; it is itself a colonial inheritance, one in which those with the power to shape economic and political structures also claim the authority to define what counts as legitimate ethical knowledge, and in which Africans are treated as objects rather than authors of responsible AI. We conclude by saying that answering the question of whose responsibility is embedded is significantly aligned with the decolonial work of contesting who gets to define responsibility in the first place.

As new meanings of responsible AI emerge from Africa, it is important to note that the critical work of identifying coloniality is not a one-off diagnostic exercise but a continuous interrogation of the assumptions that underlie the concept. African scholars are actively engaged in unpacking the internal contradictions, exclusions, and power dynamics within responsible AI, not to reject the concept outright, but to reveal how contingent, rather than universal, its dominant meaning actually is. This is not a method with fixed steps, but a practice of ongoing questioning that decolonial theorists insist has no final closure: coloniality is not dismantled by a single act of critique any more than colonialism was fully undone by formal independence. In the context of responsible AI, this critical work becomes a tool to examine the colonial, and often gendered, assumptions embedded in dominant technological narratives, allowing for the emergence of alternative, pluriversal understandings.

In this paper, we have shown that this critical, decolonial phase involves questioning how AI lifecycles are shaped by coloniality, extractivism, and epistemic injustice. It calls into question the dominance of Global North data practices and models, computational logics, and ethical frameworks that often neglect African ethical traditions. Whether by highlighting the environmental harms of AI infrastructures, critiquing linguistic and cultural gaps, or resisting algorithmic colonialism, African scholars are playing a major role in this critical, decolonial work.

However, critique and the constructive efforts currently available are not enough on their own. The move toward construction or reconstruction requires imaginative policy and regulatory frameworks rooted in African epistemologies, relational ethics, and community-defined values. This includes drawing from frameworks such as Ubuntu, Ujamaa and Umunna, as well as indigenous knowledge systems, ecofeminist thought, and intersectional justice, to inform the design, deployment, use and governance of AI. It also involves practical approaches to achieving responsible AI that is aligned to African values and that foreground collective wellbeing. What effective practical approaches can help embed African values in practice?

This critical exposé of the concept of responsible AI demonstrates that we need to move beyond a universalist framing of ethical issues, mitigation strategies, and normative assumptions in AI, toward a pluriversal understanding that acknowledges diverse cultural, epistemic, and socio-political realities. This shift challenges the dominance of Global North-centric narratives and frameworks in AI ethics, and instead invites engagement with multiple, co-existing ways of knowing, valuing, and governing technologies, including indigenous, African, feminist, and decolonial perspectives. In our view, "responsibility" is not a fixed, technocratic standard, but a contested, context-dependent practice rooted in specific worldviews, relationalities, and historical power dynamics. A pluriversal approach necessitates much broader and more inclusive dialogues about AI ethics, bringing in voices and perspectives that have traditionally been neglected. We advocate for a radical shift in how we conceive and practice responsible AI, moving from a top-down, universalist model to a more nuanced, bottom-up, and culturally sensitive approach that truly acknowledges the global diversity of human experience.

Looking towards the future, responsible AI in Africa must be approached not as a concept with a fixed meaning, but as a living, iterative process of knowledge production and social negotiation. Future research and practice of responsible AI must place African voices not only as critics of dominant systems but as co-creators of alternative meanings or understandings. This requires sustained investment in education, infrastructure, and pan-African networks of solidarity within Africa. In AI, we must be open to a pluriversal future, where multiple ways of being, knowing, and governing technology can co-exist.

In moving from critique to construction, the challenge is not only to make AI more inclusive or diverse in its current form, but to rethink what intelligence, responsibility, and technology can mean to and for Africa. This is both an ethical imperative and an epistemic opportunity.

## Data Availability

The data supporting this study consist of the corpus of publications included in the multivocal literature review. The full list of reviewed academic and grey literature sources is provided in the Supplementary Material. As all sources are publicly available publications, no primary datasets were generated. Further details regarding search strategy, screening criteria, and selection procedures are described in the Methods section.

## Footnotes

1 This speech can be found on youtube https://www.youtube.com/live/oNP6W8bl0XI?t=13625s

2 Deep Learning Indaba https://deeplearningindaba.com/ was founded to address the low participation of Africans in AI and ML research, development, and innovation.
